# Human papillomavirus genotype and viral load agreement between paired first-void urine and clinician-collected cervical samples

**DOI:** 10.1007/s10096-017-3179-1

**Published:** 2018-02-07

**Authors:** Severien Van Keer, Wiebren A. A. Tjalma, Jade Pattyn, Samantha Biesmans, Zoë Pieters, Xaveer Van Ostade, Margareta Ieven, Pierre Van Damme, Alex Vorsters

**Affiliations:** 1 Centre for the Evaluation of Vaccination (CEV), Vaccine & Infectious Disease Institute (VAXINFECTIO), Faculty of Medicine and Health Sciences, University of Antwerp, Universiteitsplein 1, Wilrijk (Antwerp), 2610 Belgium; 2 Multidisciplinary Breast Clinic, Unit Gynaecologic Oncology, Department of Obstetrics and Gynaecology, Antwerp University Hospital (UZA), Edegem, Belgium; 3 Molecular Imaging, Pathology, Radiotherapy, Oncology (MIPRO), Faculty of Medicine and Health Sciences, University of Antwerp, Wilrijk (Antwerp), Belgium; 4 Centre for Statistics, I-Biostat, Hasselt University, Hasselt, Belgium; 5 CHERMID; Vaccine & Infectious Disease Institute (VAXINFECTIO), Faculty of Medicine and Health Sciences, University of Antwerp, Wilrijk (Antwerp), Belgium; 6 Laboratory of Proteinscience, Proteomics & Epigenetic Signalling (PPES), Faculty of Pharmaceutical, Biomedical and Veterinary Sciences, University of Antwerp, Wilrijk (Antwerp), Belgium; 7 Laboratory of Medical Microbiology (LMM); Vaccine & Infectious Disease Institute (VAXINFECTIO); Faculty of Medicine and Health Sciences, University of Antwerp, Wilrijk (Antwerp), Belgium

## Abstract

The performance and acceptability of first-void urine as specimen for the detection of HPV DNA in a Belgian referral population was evaluated using an optimized sample collection and processing protocol. One hundred ten first-void urine and cervical samples were collected from 25- to 64-year-old women who were referred for colposcopy (January–November 2016). Paired samples were analyzed by the Riatol qPCR HPV genotyping assay. Acceptability data were gathered through questionnaires (NCT02714127). A higher high-risk HPV DNA prevalence was observed in first-void urine (*n* = 76/110) compared to cervical samples (*n* = 73/110), with HPV31 and HPV16/31 being most prevalent correspondingly. For both any and high-risk HPV DNA, good agreement was observed between paired samples (Cohen’s Kappa of 0.660 (95% CI: 0.486–0.833) and 0.688 (95% CI: 0.542–0.835), respectively). In addition, significant positive correlations in HPV copies (per microliter of DNA extract) between paired samples were observed for HPV16 (r_s_ = 0.670; FDR (false discovery rate)-adjusted *p* = 0.006), HPV18 (r_s_ = 0.893; FDR-adjusted *p* = 0.031), HPV31 (r_s_ = 0.527; FDR-adjusted *p* = 0.031), HPV53 (r_s_ = 0.691; FDR-adjusted *p* = 0.017), and HPV68 (r_s_ = 0.569; FDR-adjusted p = 0.031). First-void urine sampling using a first-void urine collection device was preferred over a clinician-collected cervical sample. And mostly, first-void urine sampling at home was favored over collection at the clinic or the general practitioner’s office. First-void urine sampling is a highly preferred, non-invasive method that ensures good agreement in HPV DNA (copies) with reference cervical samples. It is particularly interesting as a screening technique to reach non-participants, and its clinical performance should be further evaluated.

**Electronic supplementary material:**

The online version of this article (10.1007/s10096-017-3179-1) contains supplementary material, which is available to authorized users.

## Introduction

To date, cervical cancer (CxCa) remains a significant problem worldwide, representing the fourth most common cancer in women [1]. The identification of human papillomavirus (HPV) as the principal cause of CxCa and the ongoing improvement of diagnostic tools leading to high-throughput screening have changed the paradigm of CxCa prevention. Prophylactic HPV vaccines have been introduced that protect millions of women against HPV16/18, and recently, HPV16/18/31/33/45/52/58 associated CxCa and genital warts (HPV6/11) [2, 3]. HPV-based screening is currently of high interest, providing 60–70% greater protection against invasive CxCa compared to cytology-based screening [4, 5]. Nevertheless, challenges remain to attaining better uptake in screening programmes, as well as more accurate detection of HPV and triage markers in self-collected specimens [6]. These challenges can be diverse, and include practical, emotional, and cognitive barriers [7, 8]. HPV DNA testing on a self-sample has been proposed as an additional strategy to reach non-attendees [9, 10]. The impact of offering vaginal/cervical- [11–13] and urine-based [14] self-sampling to increase participation and screening coverage has become quite apparent. Non-invasive urine sampling has been recognized as the preferred choice for self-sampling compared to the currently available methods that involve the collection of vaginal/cervical material [15–17].

The use of first-void urine (FVU) as a liquid biopsy for HPV DNA testing is promising. High correlations have been established between urinary HPV DNA and cervical infections [18–28]. The theory put forward for identifying HPV DNA in the urine of women with vaginal or cervical HPV infections is based on the fact that during urination, the first part of the urine void (defined as *first-void urine*) captures mucus and debris from exfoliated cells from the female genital organs, including the cervix. This explains why the first collected part of a urine void contains significantly more HPV DNA than the subsequent part [25, 29]. There is also some remaining confusion regarding the definition of FVU, as it is often mistaken for the first urine of the day [29]. Adding a nucleic acid preservative to urine samples has proven effective to prevent nucleases from degrading cell-associated and cell-free DNA during transport, storage, and pre-analytical processing steps [30, 31]. In the last decade, the use of HPV type-specific copy number measurement in cervical samples (CS) for clinical management of HPV infections has been reported. However, the clinical utility of this method to differentiate between cervical precancerous grades remains challenging [32–37]. This raises the question (i) as to whether testing of (first-void) urine lacks the sensitivity and the ability to correlate between HPV copy number and a cervical precancerous state [38, 39], or (ii) whether a high concordance in HPV copy number exists between paired urine and CS [24], and (iii) if a correlation exists between HPV copy number and cervical precancerous state [23].

This study was designed as a pilot to investigate the presence of biomarkers in FVU for CxCa screening and triage of HPV-infected women, including HPV copy number as a candidate biomarker of interest. Therefore, HPV genotype agreement observed between paired FVU and CS was assessed using an optimized protocol for FVU collection, storage, and pre-analytical processing, in combination with a qPCR-based assay. In the present study, the HPV prevalence and (genotype) concordance between paired FVU and CS in a Belgian colposcopy cohort was investigated. Furthermore, we assessed the HPV copy number agreement between paired samples. Finally, we report on the acceptability of FVU sampling using a FVU collection device.

## Materials and methods

### Study participants

**Fig. 1:**
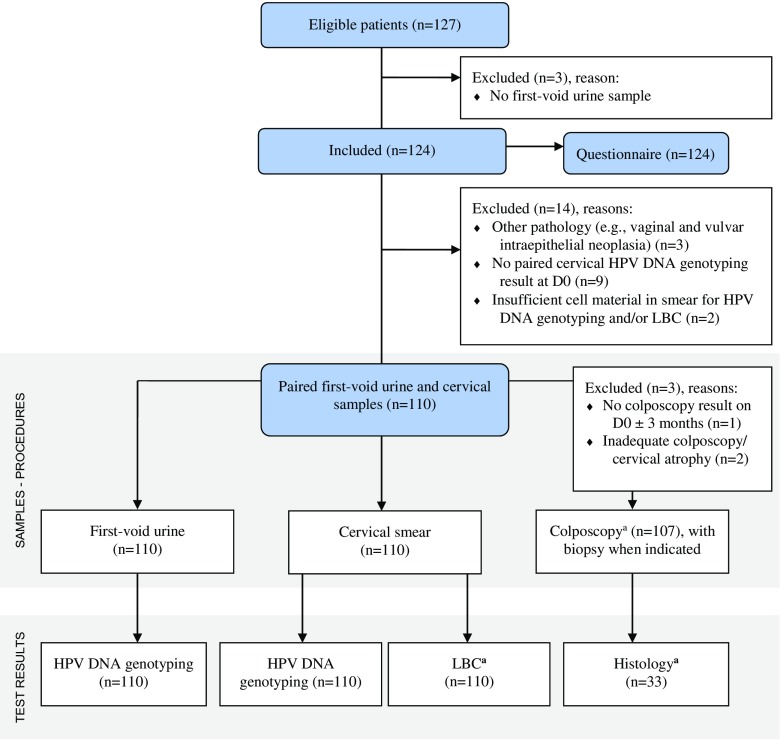
Flow diagram for the inclusion of study participants, samples, and medical records. All 127 eligible patients received a questionnaire containing, next to personal, inquiries about the acceptability of first-void urine (FVU) collection. Surveys from 124 out of 127 women who provided a Colli-Pee® (Novosanis, Belgium) collected FVU sample were included to investigate acceptability. ^a^When unavailable at D0 (day of study visit/FVU collection), the colposcopy, LBC (liquid based cytology), and histology results from D0 ± 3 months were included for data analysis instead. Thus, two additional colposcopy results were included, as well as one LBC and six histology results. No results were included from D0 ± 3 months if the woman underwent surgical treatment for high-grade cervical abnormalities in this period

Women (aged 25–64) who were referred to the colposcopy clinic at the Antwerp University Hospital (UZA, Belgium) were screened for eligibility during January and November 2016 (NCT02714127). The criteria included referral due to abnormal cytology or infection with one or multiple HPV genotypes. Patients who underwent cervical conisation in the previous year were excluded. Women who fit the criteria were informed about the study and received an information brochure beforehand. Paired FVU and cervical HPV DNA genotyping results were available for 110 out of 127 eligible patients (Fig. 1). All procedures performed in studies involving human participants were in accordance with the ethical standards of the institutional and/or national research committee (UZA/University of Antwerp, Belgium (B300201525585)) and with the 1964 Helsinki declaration and its later amendments or comparable ethical standards. Informed consent was obtained from all individual participants included in the study. To ensure patient confidentiality, each study participant received a personal identifier number to which all data were linked.

### Sample collection, processing, and storage

Upon arriving at the clinic, informed consent was obtained, and it was explained to women how to collect the FVU sample. Standard, illustrated instructions provided by the device company were also available. Women were asked to collect a FVU sample with the Colli-Pee*®* device (Novosanis, Belgium) prior to their visit with the gynecologist for a CS and colposcopy. Women were requested beforehand to not extensively wash their genitals before the visit at the clinic and to not urinate at least 1 h prior to this visit. Upon collection, FVU samples were immediately placed on ice and transported to the UZA laboratory, where they were aliquoted and preserved on dry ice before storage at −80 °C. For HPV DNA genotyping, one volume of urine conservation medium (UCM) [31] was added to two volumes of FVU before storage at −80 °C.

Data from CS (HPV DNA genotyping, liquid based cytology (LBC)) and colposcopy (with an optional biopsy) from D0 (day of the study visit) ± 3 months were retrieved from the medical records of each study participant. CS were collected with a Cervex-Brush® (Rovers Medical Devices, The Netherlands), which were transferred in 20 ml PreservCyt® collection medium (Hologic Europe, Belgium) and analyzed with the ThinPrep® Pap Test (Hologic Europe). During colposcopy, the cervix was visually inspected for abnormalities and graded as normal, low-, or high-grade cervical abnormality. When indicated, a biopsy for histological confirmation was taken. Women scheduled for an ambulant conisation were also included, as were their histology results from the conus. Both LBC and histology were performed at the UZA pathology laboratory and were graded according to the Bethesda and Cervical Intraepithelial Neoplasia (CIN) classifications, respectively. Women graded with different colposcopic and histological outcomes were classified according to the most severe stage. CS were taken, and colposcopies were performed by two colposcopists.

### DNA extraction of first-void urine samples

UCM-buffered FVU aliquots were thawed (after storage for 2–12 months) and DNA extraction was performed with an in-house protocol [31]. Briefly, 4 ml of the aliquot was centrifuged at 3820 g for 20 min at 20 °C in an Amicon Ultra-4 50 K filter device (Merck Millipore, Belgium). Following filtration, 2 ml of NucliSENS® Lysis Buffer (BioMérieux, Benelux) was added to the concentrate retained on the filter and incubated for 10 min at ambient temperature. The solution retained on the filter was subsequently transferred to the NucliSENS Lysis Buffer vial before DNA extraction using NucliSENS® easyMAG® (BioMérieux, Benelux). DNA was eluted in 55 μl, after which 35 μl was transferred to a second vial with elution buffer (BioMérieux, Benelux) to reach a total volume of 75 μl of DNA extract used for HPV DNA genotyping.

### HPV DNA genotyping

#### Cervical samples

HPV DNA genotyping of CS was performed with the Riatol qPCR HPV genotyping assay (Riatol assay), quantifying 12 high-risk (HR) (HPV16/18/31/33/35/39/45/51/52/56/58/59), one probable HR (HPV68), three possible HR (HPV53/66/67), and two low-risk (LR) (HPV6/11) HPV genotypes [40] and β-globin as described elsewhere [41, 42]. Briefly, 400–800 μl of the remaining LBC specimens were subjected to automatic nucleic acid preparation. Following extraction, HPV and β-globin were quantified using highly sensitive multiplex qPCR (LightCycler® 480, Roche, Switzerland), with 1–100 HPV copies detected per reaction (copies/μl). β-globin was amplified to assess the DNA quality and to determine the number of cells present in the sample. HPV DNA positivity was reported in HPV copies per cell by dividing the HPV copies per microliter (µl) DNA extract by the number of cells per µl. The positivity threshold was set at 0.0001 HPV copies per cell [42, 43]. HPV copies per cell, hereafter referred to as the human DNA (hDNA) equivalent, was calculated back to HPV copies per µl of DNA extract to enable comparison with FVU HPV DNA results.

#### First-void urine

HPV DNA genotyping using FVU DNA extracts was performed with the Riatol assay [41, 42] according to an optimized protocol for FVU [44]. The difference between the protocol used for CS and FVU was that, for the latter, DNA extracts were directly pipetted into the 96-well plate, followed by qPCR, bypassing the sample preparation and DNA extraction steps. FVU samples were subjected to batches in random order for DNA extraction and HPV DNA genotyping. The HPV DNA results were reported in copies per hDNA equivalent and per microliter of DNA extract for each genotype separately. The concentration of human and HPV DNA in nanogram (ng) and copies per µl of DNA extract was 44.08 times lower than in 1 ml of the original urine fraction. Thus, HPV DNA copies per µl of DNA extract should be multiplied by this factor or 1.64 logarithm-to-base 10 should be added to the log10 copies to determine human and HPV DNA copies per milliliter of FVU.

### Statistical analysis

The percent correct and Cohen’s Kappa (κ) were calculated to assess the HPV genotype agreement between paired samples. In addition, the McNemar’s odds ratio (OR) and test were computed to compare the association between FVU or CS for a given HPV genotype. In case a zero cell count occurred, a constant of 1 was added to each cell in the table [45]. The Wilcoxon matched pairs signed rank test was used to test for differences in dependent continuous measures and for differences within levels of categorical measures. hDNA and HPV copy number correlations were calculated using Pearson and Spearman rank correlations, respectively. All *p*-values were corrected for multiple testing using the false discovery rate (FDR) method [46]. Statistical analyses were performed at a significance level of 5% using the statistical software R version 3.3.3 [47] and IBM SPSS Statistics Version 24. Additional graphs were generated using Excel Microsoft Office Professional Plus 2016 and JMP Pro 13.

## Results

### Study population

The median participant age in our study population was 36 (interquartile range (IQR): 29–44 years old), with a gradual decrease in the number of participants per increasing age group. LBC and HR-HPV results are detailed in Table 1 according to age group. Colposcopy results were available from 107 out of 110 women, with 29.91% classified as normal (*n* = 32/107), 52.34% with low-grade (*n* = 56/107), and 17.76% with high-grade cervical abnormality (*n* = 19/107). Histology results were available from 33 women and revealed that 33.33% of women had cervical intraepithelial neoplasia grade 1 (CIN1) (*n* = 11/33), 18.18% with CIN2 (*n* = 6/33), 27.27% with CIN3 (*n* = 9/33), and 21.21% with no proven CIN (*n* = 7/33).

**Table 1:** Prevalence of HR-HPV and the liquid-based cytology stage according to age group

Age group (years)	Number of women (%)	Sum of HR-HPV^a^ positive samples (%)		Liquid based cytology grade (%)					
		First-void urine (%)	Cervical (%)	NILM	AGC	ASC-US	LSIL	ASC-H	HSIL
25–29	33 (30.00)	25 (75.76)	25 (75.76)	15	0	6	4	4	4
30–34	19 (17.27)	11 (57.89)	10 (52.63)	11	1	2	5	0	0
35–39	16 (14.55)	8 (50.00)	11 (68.75)	9	0	1	4	1	1
40–44	16 (14.55)	15 (93.75)	12 (75.00)	9	0	2	2	2	1
45–49	9 (8.18)	7 (77.78)	7 (77.78)	3	0	3	2	1	0
50–54	10 (9.09)	6 (60.00)	5 (50.00)	7	0	1	1	0	1
55–59	3 (2.73)	2 (66.67)	2 (66.67)	1	0	2	0	0	0
60–64	4 (3.64)	2 (50.00)	1 (25.00)	3	0	1	0	0	0
*Total (%)*	*110 (100.00)*	*76 (69.09)*	*73 (66.36)*	*58 (52.73)*	*1 (0.91)*	*18 (16.36)*	*18 (16.36)*	*8 (7.27)*	*7 (6.36)*

*NILM* negative for intraepithelial lesion and malignancy, *AGC* atypical glandular cells of undetermined significance, *ASC-US* atypical squamous cells of undetermined significance, *ASC-H* atypical squamous cells, cannot exclude HSIL, *LSIL* low-grade squamous intraepithelial lesion, *HSIL* high-grade squamous intraepithelial lesion

^a^High-risk (HR) HPV types: HPV16/18/31/33/35/39/45/51/52/56/58/59 [40]

### HPV genotype prevalence and concordance

The use of a FVU collection device (Colli-Pee*®*) resulted in a median collection volume of 19 ml FVU (IQR: 18–19 ml), collected within a median time span of 1 h and 56 min (IQR: 1:23–2:50) after the previous urination. Samples were frozen in aliquots after the addition of a DNA preservative (UCM) within a median time span of 12 min (IQR: 0:11–0:16) after urination. No invalid hDNA test results (β-globin negative) were reported in the 110 included CS, nor in FVU after in-house ultrafiltration and DNA extraction.

**Fig. 2:**
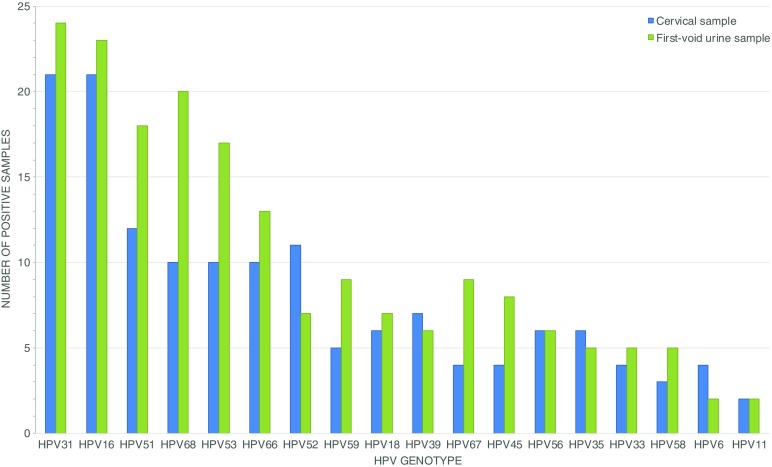
Prevalence of HPV genotypes according to sample type. The sum of cervical (CS; *blue bars*) and first-void urine samples (FVU; *green bars*) observed as positive for HPV are separately displayed on the y-axis for each genotype (x-axis). Ranking of HPV genotypes was performed according to the combined number of CS and FVU samples that tested positive

The results of the Riatol assay in paired FVU and CS demonstrated an HPV prevalence of 80.00 (*n* = 88/110) and 75.45% (*n* = 83/110) in FVU and CS, respectively, with a good κ-agreement of 0.660 (95% CI: 0.486–0.833). The same pattern was observed for HPV16/18 and HR-HPV DNA, with a prevalence of 25.45 and 69.09% in FVU, and 23.64 and 66.36% in CS, respectively. A very good and good agreement for HPV16 and/or 18 (κ: 0.902; 95% CI: 0.807–0.996) and HR-HPV DNA (κ: 0.688; 95% CI: 0.542–0.835) between paired samples was observed, with a McNemar OR of 3.000 (95% CI: 0.241–157.492; FDR-adjusted *p* = 0.822) (n_total_ = 110; *n* = 25 FVU+/CS+, *n* = 3 FVU+/CS-, *n* = 1 FVU-/CS+, *n* = 81 FVU-/CS-) and 1.500 (95% CI: 0.477–5.121; FDR-adjusted p = 0.822) (n_total_ = 110; *n* = 67 FVU+/CS+, *n* = 9 FVU+/CS-, n = 6 FVU-/CS+, *n* = 28 FVU-/CS-), respectively (Online Resource 1). HPV31 (FVU) and HPV16 and 31 (CS) were observed to be most prevalent in our referral population, followed by HPV16 and 68 for FVU, and HPV51 and 52 for CS (Fig. 2).

**Fig. 3:**
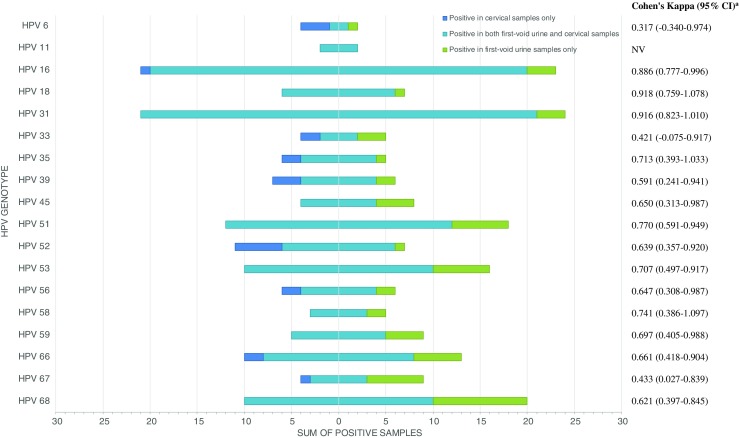
HPV genotype distribution and agreement of paired cervical (CS) and first-void urine samples (FVU). The number of samples that tested positive in both FVU and CS are indicated by turquoise bars. Discordant samples for each genotype are separately indicated by *blue* (CS only) and *green* (FVU only) *bars*. ^a^The Cohen’s Kappa (κ) was judged as follows: κ ≤ 0.20, poor; 0.21 ≤ κ ≤ 0.40, fair; 0.41 ≤ κ ≤ 0.60, moderate; 0.61 ≤ κ ≤ 0.80, good; and κ ≥ 0.81, very good agreement [48]. *95% CI* 95% confidence interval, *NV* Due to the zero cell count in the discordant pairs for HPV11, κ was not calculated

Overall, a (very) good agreement was observed at the genotype level, except for HPV33/39/67 and 6, where moderate and fair κ-agreements were observed, respectively (Fig. 3). The observed odds of having a positive result for a single infection for FVU versus CS was 0.263 (95% CI: 0.077–0.729, FDR-adjusted *p* = 0.041) (CS: *n* = 45/92; FVU: *n* = 31/92). In contrast, the observed odds of having a positive result for FVU versus CS was 7-fold higher in the case of multiple infections (McNemar OR: 7.333, 95% CI: 2.203–38.269, FDR-adjusted *p* = 0.004) (CS: *n* = 38/92; FVU: *n* = 57/92) (Online Resource 1), with a maximum of five types simultaneously detected in CS as opposed to eight in FVU.

### HPV copy number agreement

For all 110 paired samples, β-globin was amplified to determine the amount of hDNA per µl of DNA extract. The median hDNA concentration in FVU and CS was 14.68 (IQR: 6.57–31.08) and 67.79 (IQR: 32.53–117.63) ng per µl of DNA extract, respectively. On the log scale, we detected 0.67 log more of hDNA in CS (1.83 ± 0.04 SE (standard error)) compared to FVU (1.16 ± 0.05 SE) on average. No linear correlation (Pearson) was observed between log hDNA and age for CS (0.043, FDR-adjusted *p* = 0.654) nor FVU (−0.126, FDR-adjusted *p* = 0.382).

Significant positive Spearman rank correlations (r_s_) in HPV copies per µl of DNA extract between paired samples were observed for HPV16 (r_s_ = 0.670; FDR-adjusted *p* = 0.006), HPV18 (r_s_ = 0.893; FDR-adjusted *p* = 0.031), HPV31 (r_s_ = 0.527; FDR-adjusted p = 0.031), HPV53 (r_s_ = 0.691; FDR-adjusted *p* = 0.017), and HPV68 (r_s_ = 0.569; FDR-adjusted p = 0.031) (Online Resource 2). Correlations for the two most carcinogenic HPV types (HPV16 and 18) are illustrated in Online Resource 3.

**Fig. 4:**
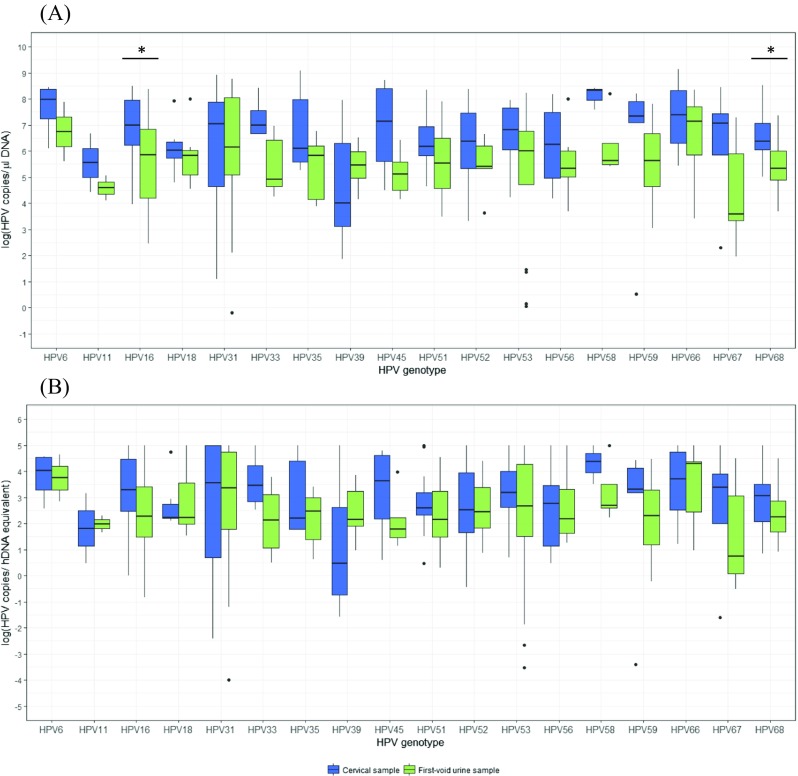
Distribution of log transformed HPV copies in paired cervical (CS) and first-void urine samples (FVU). The log transformed HPV copies per (**A**) microliter of DNA extract and (**B**) per hDNA equivalent in CS (*blue bars*) and FVU (*green bars*) are displayed on the y-axis for each genotype (x-axis). Significantly different median log HPV copies between paired samples are indicated by an asterisk (Wilcoxon matched pairs signed rank test), when *p*-values adjusted for multiple testing using the false discovery rate (FDR) were smaller than 0.05

No significant Spearman’s rank correlations were observed between HPV copies per µl of DNA extract (for each individual genotype), and the number of cells present in either CS or FVU. For comparison, the log transformed HPV copies per µl of DNA extract and per hDNA equivalent for each individual genotype are represented in Fig. 4 (absolute numbers and statistics are detailed in Online Resource 3). A higher median log HPV copy number was observed in 12 out of 13 and 11 out of 13 (probable) HR-HPV genotypes in CS versus FVU samples reported in HPV copies per µl of DNA extract and per hDNA equivalent, respectively. These differences were only significant for HPV16 and 68, for which a 1.134 (FDR-adjusted *p* = 0.040) and 1.017 log (FDR-adjusted p = 0.040) increase in the median copy number per µl of DNA extract was observed in CS versus FVU, respectively.

### Acceptability of first-void urine sampling

Acceptability data were gathered through questionnaires completed by 124 eligible women after providing a Colli-Pee*®*-collected FVU sample. The majority of the participants favored FVU sampling (*n* = 83/113; 73.45%) over a clinician-collected sample (*n* = 24/113; 21.24%). Five out of 83 women preferred the Colli-Pee® method if it were as reliable as a smear collected by a doctor/gynecologist. One woman only preferred this method if it could be performed at home. Another woman brought up the issue that no questions could be asked to the doctor/gynecologist. Although ‘no preference’ was not an option in the questionnaire, five out of 113 women (4.42%) wrote down that they did not have a preference between the two methods or checked both boxes. One woman (*n* = 1/113; 0.88%) preferred yearly FVU collection with the Colli-Pee® and bi-yearly smear collection by the doctor/gynecologist. In addition, from the data that was collected, FVU collection at home was preferred over collection at the clinic or the general practitioner’s office. This was demonstrated by 115 out of 122 (94.26%) and 81 out of 120 (67.50%) of women agreeing that FVU sampling was a good method to perform at home versus at the clinic or the general practitioner’s office, respectively (Online Resource 5).

## Discussion

Several studies have explored the use of urine sampling as a useful and non-invasive alternative for HPV detection for screening purposes [18–28, 49–52], and to reach screening non-attendees via home-based self-sampling [14]. However, these results are not conclusive due to a number of variables that could negatively affect HPV detection in urine. In this study, we investigated the usefulness of FVU as a specimen for the detection of HPV DNA among women referred for colposcopy using an optimized sample collection and processing protocol and the results obtained from CS as references.

Key notes for improved human and HPV DNA detection in urine have been studied [16, 17, 21, 25, 26, 30, 31] and were recently summarized [6]. Briefly, optimized urinary HPV DNA detection should include: (i) use of FVU; (ii) prevention of human/HPV DNA degradation during extraction and storage by adding a preservative; (iii) processing of a sufficient volume of whole urine; and the (iv) use of an analytically sensitive HPV test. In this study, FVU samples were collected prior to the clinical exam using a FVU collection device to ensure the collection of a standardized FVU volume (19 ml; IQR: 18–19 ml). In addition, women were asked to not extensively wash their genitals and to not urinate at least 1 h prior to sample collection. The suitability of the optimized techniques used, namely, ultrafiltration and automated extraction of DNA from preserved, unfractionated FVU samples [21, 31], was confirmed by the presence of hDNA in all FVU samples. This was in contrast to other studies in which invalid test results were reported [14, 24, 38]. The validity of hDNA as indicator for proper sample storage, processing, and detection of HPV DNA has been questioned [31] and was discussed in a previous study [22]. Still, samples testing negative for hDNA might point towards suboptimal collection, storage, and (pre-)analytical processing protocols. In addition, the HPV test used is a highly sensitive qPCR-based assay employed by the Belgian HPV reference laboratory.

This study provided evidence of a good HR-HPV agreement between paired FVU and CS (κ = 0.688; 95% CI: 0.542–0.835). Furthermore, very good agreement was observed for HPV16, 18, and 31, with no significant difference in the observed odds of FVU and CS positive for the abovementioned genotypes, nor for HR-HPV. Where others reported a lower HR-HPV prevalence in urine samples compared to cervical specimens [38], we observed an HR-HPV prevalence of 69 (*n* = 76/110) and 66% (*n* = 73/110) in FVU and CS, respectively. These results are in line with studies where optimized collection and pre-analytical processing protocols were applied [19, 20, 22, 31, 50]. Previous observations made in a similar study performed by Bissett and colleagues (2011) [38], who collected 253 paired urine and CS from women referred to colposcopy, reported an HR-HPV prevalence of 70 and 81%, respectively. The somewhat lower HR-HPV prevalence observed in our study could be because our study population consisted of a greater number of women diagnosed with normal cytological findings. In a screening setting, a lower median age of the women could result in a higher HR-HPV prevalence [14, 23]. However, no decreasing trend of HR-HPV prevalence with increasing age was observed in our referral population. Consistent with Belgian data, in which HPV16 and 31 are ranked as two out of three most prevalent genotypes in women with normal, low- and high-grade abnormal cytology [53] and invasive CxCa [54, 55], both HPV16 and 31 (CS) and HPV31 (FVU) were the most prevalent genotypes observed in our study population. These results are in agreement with previous observations made in a referral population [38]. Furthermore, we observed that in samples positive for any HPV genotype in at least one of the paired samples, more single-type infections were observed in CS, whereas multiple-type infections were more frequent in FVU. In the CapU study, a large proportion of multiple urinary infections (44.80%) was observed as well [14]. The discrepancy in the HPV results in our cohort was primarily evident in HPV genotypes classified as probable and possible high-risk. These results may suggest that FVU might sample next to cervical, also more infections originating from the vagina and vulva.

The HPV viral load in CS is typically defined as the number of HPV copies per cell, herein referred to as the hDNA equivalent. As similarly reported by Payan and colleagues [24], no significant correlation was observed in our study between HPV copies per µl of DNA extract and the number of cells per sample. Additionally, no difference can be made between HPV-infected and non-infected cells. Because we were interested in detecting the total amount of HPV DNA (cell-free and -associated) in FVU per sample volume, and since HPV DNA in urine originates from mucus and debris from exfoliated cervical cells, unlike clinician-collected samples, where cervical cells are scraped off the cervix, we did not normalize HPV copy number according to sample cellularity. This enabled comparison of the FVU and cervical HPV DNA results. However, this calculation does not take stratification according to sample cellularity during DNA extraction into account [22], which is performed on CS prior to the Riatol assay [42]. Thus, HPV copies are reported per µl of DNA extract, and not per volume of initial sample collected. Furthermore, no significant difference in the median HPV copy number (per µl of DNA extract) was observed between paired samples, except for HPV16 and 68. The trend towards good agreement between FVU and CS for HPV DNA (per µl of DNA extract) was also observed by the significantly positive correlations observed between paired samples for HPV16, 18, 31, 53, and 68.

Lastly, the results obtained from the questionnaires confirmed data from previous studies [15, 16] reporting that (first-void) urine collection is the most preferred sampling method for CxCa screening by women with respect to vaginal/cervical self-sampling and clinician collected samples. We observed that FVU collection using a FVU collection device (Colli-Pee®) was preferred over a clinician-collected CS (*n* = 83/113 (73%) favored FVU sampling). The acceptability of FVU sampling was also described by Leeman and colleagues, comparing the preference of a clinician-collected CS, vaginal self-sample (Evalyn brush™, Rovers Medical Devices, the Netherlands), and FVU sample (Colli-Pee®) in 91 women providing all three samples, with an overall rating of 7.6, 8.1, and 8.6 out of 10, respectively [17]. Notably, FVU collection at home was preferred over collection at the clinic or the general practitioner’s office. This is in agreement with 87% of women (referral population) who reported being comfortable receiving a urine collection package at home in a study by Senkomago and colleagues (2016) [16]. Together with results from the CapU study, in which home-based urine sampling increased the participation rate by 11–16% in screening non-responders (40–65 years) after receiving two reminders [14], these data indicate that FVU sampling shows potential as liquid biopsy source to reach (non-attending) women for home-based CxCa screening. Nevertheless, it remains to be investigated how home-based FVU sampling can be ideally implemented in practice, since in contrast to the positive outcomes obtained in the studies mentioned above, a lack of privacy at home, education, place of residence (urban versus rural), and women’s concerns about collecting the self-sample properly themselves could influence the acceptability of self-sampling [56]. The use of urine sampling to monitor the impact of HPV-vaccination has also been reported, focusing on urine as a sample to assess viral endpoints [20, 57, 58]. The World Health Organization and International Agency for Research on Cancer have already reported on a vaccine-impact study using viral endpoints in FVU in approximately 2,000 young women [20], demonstrating that FVU is a well-accepted liquid biopsy source that can be easily and non-invasively collected in large-scale studies.

Limitations of our study need to be acknowledged, most notably the relatively small sample size. As our study was not designed to investigate HPV copy number trend and lesion severity, or to compare the association of FVU and CS for individual HPV-genotypes, one should be cautious with interpreting the results. The acceptability results should be interpreted with caution, as several women wrote down that they had no preference between the two methods, while the option ‘no preference’ was not given in the questionnaire. As this could bias our results, we choose to descriptively report the acceptability data obtained in this study. In addition, despite the heterogeneous character of our study population (including women redirected to colposcopy for the first time, or women who had been followed-up for a long time, whether after treatment or not), participating women were well-characterized. The absence of a home-based setting, which is the purpose of using this liquid biopsy method [6], will be assessed in an adjoining study. As this was a pilot study to investigate the presence of biomarkers in FVU, including HPV DNA (copy number) and to develop analytical protocols, no preservation buffer was prefilled in the collector vials to avoid incompatibilities with other assays under research. Still, samples were immediately placed on ice after collection and stored with preservation buffer within a median time span of 12 min (IQR: 0:11–0:16) to reduce the loss of human and HPV DNA to a minimum. At last, a higher disease prevalence in referral populations might benefit from an accurate measure of sensitivity at the cost of less reliable specificity measures.

In conclusion, in this study, we observed that, when appropriately sampled, stored, and processed, testing FVU for (HR-) HPV DNA ensured a good agreement with CS in a Belgian referral population. At the genotype level, significant positive correlations were observed between paired samples for the three most carcinogenic HPV types in our country, HPV16, 18, and 31, as well as for HPV53 and 68. The median copy number observed in paired samples did not differ except for HPV16 and 68. Furthermore, FVU sampling is highly preferred, non-invasive, and can be performed at home. This method is particularly interesting as a screening tool to overcome the hurdle of screening among non-responders. However, further research is imperative to standardize the procedures involved in HPV DNA detection in FVU and to evaluate its clinical performance in large population-based studies.

## Electronic supplementary material

ESM 1 (DOCX 140 kb)
