# Supplementary material for: Human papillomavirus genotype and viral load agreement between paired first-void urine and clinician-collected cervical samples
Source: Eur J Clin Microbiol Infect Dis. 2018 Feb 7;37(5):859–69. doi: 10.1007/s10096-017-3179-1 (PMC5916996; doi:10.1007/s10096-017-3179-1)
Supplement: Supplementary file 1 — (DOCX 140 kb) [file 10096_2017_3179_MOESM1_ESM.docx]

Online resources belonging to an original article submitted to ‘European Journal of Clinical Microbiology & Infectious Diseases’.

**Human papillomavirus genotype and viral load agreement between paired first-void urine and clinician-collected cervical samples**

Severien Van Keer (ORCID:0000-0003-1842-7478)^1,^*, Wiebren A. A. Tjalma (ORCID:0000-0002-6618-045X)^2^, Jade Pattyn (ORCID:0000-0002-4538-9027)^1^, Samantha Biesmans^1^, Zoë Pieters^3^, Xaveer Van Ostade (ORCID:0000-0003-4078-3014)^4^, Margareta Ieven^5^, Pierre Van Damme (ORCID:0000-0002-8642-1249)^1^, Alex Vorsters (ORCID:0000-0002-0265-058X)^1^

*Corresponding author: Severien Van Keer; Campus Drie Eiken, Building R2, Universiteitsplein 1, 2610 Wilrijk (Belgium); Tel. +32(0)3/265.25.38; Fax +32(0)3/265.26.40; [severien.vankeer@uantwerpen.be](mailto:severien.vankeer@uantwerpen.be)

^1^Centre for the Evaluation of Vaccination (CEV); Vaccine & Infectious Disease Institute (VAXINFECTIO); Faculty of Medicine and Health Sciences; University of Antwerp (Belgium)

^2^Multidisciplinary Breast Clinic, Unit Gynaecologic Oncology; Department of Obstetrics and Gynaecology; Antwerp University Hospital (UZA) (Belgium); Molecular Imaging, Pathology, Radiotherapy, Oncology (MIPRO); Faculty of Medicine and Health Sciences; University of Antwerp (Belgium)

^3^CHERMID; Vaccine & Infectious Disease Institute (VAXINFECTIO); Faculty of Medicine and Health Sciences; University of Antwerp (Belgium); Centre for Statistics; I-Biostat; Hasselt University (Belgium)

^4^Laboratory of Proteinscience, Proteomics & Epigenetic Signalling (PPES); Faculty of Pharmaceutical, Biomedical and Veterinary Sciences; University of Antwerp (Belgium)

^5^Laboratory of Medical Microbiology (LMM); Vaccine & Infectious Disease Institute (VAXINFECTIO); Faculty of Medicine and Health Sciences; University of Antwerp (Belgium)

**Online Resource 1. Overall HPV and genotype level agreement between first-void urine and cervical samples.**

| Cervix  (N) | First-void urine  (N) | | Agreement (%)  (95% CI) | Cohen’s  Kappa  (95% CI)^a^ | McNemar OR  (95% CI)^b^ | McNemar  p-value | FDR-adjusted  p-value^c^ |
| --- | --- | --- | --- | --- | --- | --- | --- |
|  | **Negative** | **Positive** |  |  |  |  |  |
| Any HPV |  |  |  |  |  |  |  |
| Negative | 18 | 9 | 88.18 | 0.660 | 2.250 | 0.267 | 0.606 |
| Positive | 4 | 79 | (80.82-92.96) | (0.486- 0.833) | (0.628- 9.999) |  |  |
|  |  |  |  |  |  |  |  |
| Single infection^d^ | | | | | | | |
| Negative | 42 | 5 | 73.91 | 0.475 | 0.263 | 0.007 | 0.041* |
| Positive | 19 | 26 | (64.11-81.80) | (0.294- 0.655) | (0.077- 0.729) |  |  |
|  |  |  |  |  |  |  |  |
| Multiple infection^d^ | | | | | | | |
| Negative | 32 | 22 | 72.83 | 0.478 | 7.333 | <0.001 | 0.004* |
| Positive | 3 | 35 | (62.96-80.86) | (0.304- 0.653) | (2.203- 38.269) |  |  |
|  |  |  |  |  |  |  |  |
| HPV16/18 |  |  |  |  |  |  |  |
| Negative | 81 | 3 | 96.36 | 0.902 | 3.000 | 0.625 | 0.822 |
| Positive | 1 | 25 | (91.02-98.58) | (0.807- 0.996) | (0.241- 157.492) |  |  |
|  |  |  |  |  |  |  |  |
| HPV6/11/16/18 |  |  |  |  |  |  |  |
| Negative | 78 | 3 | 94.55 | 0.860 | 1.000 | 1.000 | 1.000 |
| Positive | 3 | 26 | (88.61-97.48) | (0.750-0.969) | (0.134- 7.466 |  |  |
|  |  |  |  |  |  |  |  |
| HR-HPV^e^ |  |  |  |  |  |  |  |
| Negative | 28 | 9 | 86.36 | 0.688 | 1.500 | 0.607 | 0.822 |
| Positive | 6 | 67 | (78.71-91.56) | (0.542- 0.835) | (0.477- 5.121) |  |  |
| HPV16 |  |  |  |  |  |  |  |
| Negative | 86 | 3 | 96.36 | 0.886 | 3.000 | 0.625 | 0.822 |
| Positive | 1 | 20 | (91.02-98.58) | (0.777- 0.996) | (0.241- 157.492) |  |  |
| HPV18 |  |  |  |  |  |  |  |
| Negative | 103 | 1 | 99.09 | 0.918 | 2.000 | 1.000 | 1.000 |
| Positive | 0 | 6 | (95.03-99.95) | (0.759- 1.078) | (0.104- 117.994) |  |  |
| HPV31 |  |  |  |  |  |  |  |
| Negative | 86 | 3 | 97.27 | 0.916 | 4.000 | 0.375 | 0.781 |
| Positive | 0 | 21 | (92.29-99.07) | (0.823- 1.010) | (0.396- 196.990) |  |  |
| HPV33 |  |  |  |  |  |  |  |
| Negative | 103 | 3 | 95.45 | 0.421 | 1.500 | 1.000 | 1.000 |
| Positive | 2 | 2 | (89.80-98.04) | (-0.075- 0.917) | (0.172- 17.959) |  |  |
| HPV35 |  |  |  |  |  |  |  |
| Negative | 103 | 1 | 97.27 | 0.713 | 0.500 | 1.000 | 1.000 |
| Positive | 2 | 4 | (92.29-99.07) | (0.393- 1.033) | (0.008- 9.605) |  |  |
| HPV39 |  |  |  |  |  |  |  |
| Negative | 101 | 2 | 95.45 | 0.591 | 0.667 | 1.000 | 1.000 |
| Positive | 3 | 4 | (89.80-98.04) | (0.241- 0.941) | (0.056- 5.820) |  |  |
| HPV45 |  |  |  |  |  |  |  |
| Negative | 102 | 4 | 96.36 | 0.650 | 5.000 | 0.219 | 0.547 |
| Positive | 0 | 4 | (91.02-98.58) | (0.313- 0.987) | (0.559- 236.488) |  |  |
| HPV51 |  |  |  |  |  |  |  |
| Negative | 92 | 6 | 95.54 | 0.770 | 7.000 | 0.070 | 0.293 |
| Positive | 0 | 12 | (88.61-97.48) | (0.591- 0.949) | (0.899- 315.483) |  |  |
| HPV52 |  |  |  |  |  |  |  |
| Negative | 98 | 1 | 95.45 | 0.639 | 0.200 | 0.219 | 0.547 |
| Positive | 5 | 6 | (88.61-97.48) | (0.357- 0.920) | (0.004- 1.787) |  |  |
| HPV56 |  |  |  |  |  |  |  |
| Negative | 102 | 2 | 96.36 | 0.647 | 1.000 | 1.000 | 1.000 |
| Positive | 2 | 4 | (91.02-98.58) | (0.308- 0.987) | (0.072- 13.796) |  |  |
| HPV58 |  |  |  |  |  |  |  |
| Negative | 105 | 2 | 98.18 | 0.741 | 3.000 | 0.625 | 0.822 |
| Positive | 0 | 3 | (93.61-99.50) | (0.386- 1.097) | (0.241- 157.492) |  |  |
| HPV59 |  |  |  |  |  |  |  |
| Negative | 101 | 4 | 96.36 | 0.697 | 5.000 | 0.219 | 0.547 |
| Positive | 0 | 5 | (91.02-98.58) | (0.405- 0.988) | (0.559- 236.488) |  |  |
|  |  |  |  |  |  |  |  |
| Probable HR-HPV^e^ | | | | | | | |
| HPV68 |  |  |  |  |  |  |  |
| Negative | 90 | 10 | 90.91 | 0.621 | 11.000 | 0.006 | 0.041* |
| Positive | 0 | 10 | (84.07-94.99) | (0.397- 0.845) | (1.599- 473.475) |  |  |
|  |  |  |  |  |  |  |  |
| Possible HR-HPV^e^ | | | | | | | |
| Negative | 73 | 14 | 86.36 | 0.659 | 14.000 | 0.001 | 0.012* |
| Positive | 1 | 22 | (78.71-91.56) | (0.498- 0.819) | (2.130- 91.968) |  |  |
| HPV53 |  |  |  |  |  |  |  |
| Negative | 93 | 7 | 93.63 | 0.707 | 8.000 | 0.039 | 0.195 |
| Positive | 0 | 10 | (87.44-96.88) | (0.497- 0.917) | (1.073- 354.981) |  |  |
| HPV66 |  |  |  |  |  |  |  |
| Negative | 95 | 5 | 93.63 | 0.661 | 2.500 | 0.453 | 0.822 |
| Positive | 2 | 8 | (87.44-96.88) | (0.418- 0.904) | (0.409- 26.253) |  |  |
| HPV67 |  |  |  |  |  |  |  |
| Negative | 100 | 6 | 93.63 | 0.433 | 6.000 | 0.125 | 0.446 |
| Positive | 1 | 3 | (87.44-96.88) | (0.027- 0.839) | (0.728- 275.986) |  |  |
|  |  |  |  |  |  |  |  |
| LR-HPV^e^ |  |  |  |  |  |  |  |
| Negative | 104 | 1 | 96.36 | 0.482 | 0.333 | 0.625 | 0.822 |
| Positive | 3 | 2 | (91.02-98.58) | (-0.016- 0.980) | (0.006- 4.151) |  |  |
| HPV6 |  |  |  |  |  |  |  |
| Negative | 105 | 1 | 93.36 | 0.317 | 0.333 | 0.625 | 0.822 |
| Positive | 3 | 1 | (91.02-98.58) | (-0.340- 0.974) | (0.006- 4.151) |  |  |
| HPV11 |  |  |  |  |  |  |  |
| Negative | 108 | 0 | NV | NV | NV | NV | NV |
| Positive | 0 | 2 | NV | NV | NV | NV | NV |
|  |  |  |  |  |  |  |  |

^a^The Cohen’s Kappa (κ) was judged as follows: κ≤0.20, poor; 0.21≤ κ≤0.40, fair; 0.41≤κ≤0.60, moderate; 0.61≤κ≤0.80, good; and κ≥0.81, very good agreement [49]. ^b^The McNemar’s odds ratio (OR) was used to compare the preference of one diagnostic test over another. ^c^Significantly different proportions in HPV positivity between paired samples (McNemar’s test), with p-values adjusted for multiple testing using the false discovery rate (FDR) analysis (p<0.05 indicated by an asterisk). ^d^For single and multiple infections, cross tabulation, agreement, κ, and McNemar’s statistics were performed on samples testing positive for any HPV in either first-void urine or cervical samples (n=92/110). ^e^Classification of low (LR-) and (possible and probable) high-risk (HR-) HPV types included in the Riatol qPCR HPV genotyping assay, according to the HPV classification from the International Agency for Research on Cancer (IARC) 2012 [32]. 95% CI: 95% Confidence interval. NV: Due to the zero cell count in the discordant pairs for HPV11, no statistics were computed.

**Online Resource 2. Correlation in log HPV copies per µl DNA extract between paired first-void urine and cervical samples.**

| HPV genotype (group)^a^ | N samples positive for first-void urine and/or cervix | Spearman rank correlation | p-value | FDR-adjusted p-value^b^ |
| --- | --- | --- | --- | --- |
| HR-HPV | | | | |
| HPV16 | 24 | 0.670 | <0.001 | 0.006* |
| HPV18 | 7 | 0.893 | 0.007 | 0.031* |
| HPV31 | 24 | 0.527 | 0.008 | 0.031* |
| HPV33 | 7 | 0.393 | 0.384 | 0.466 |
| HPV35 | 7 | 0.090 | 0.848 | 0.848 |
| HPV39 | 9 | 0.196 | 0.614 | 0.696 |
| HPV45 | 8 | 0.457 | 0.255 | 0.333 |
| HPV51 | 18 | 0.466 | 0.051 | 0.124 |
| HPV52 | 12 | -0.116 | 0.720 | 0.765 |
| HPV56 | 8 | 0.482 | 0.227 | 0.322 |
| HPV58 | 5 | 0.667 | 0.219 | 0.322 |
| HPV59 | 9 | 0.609 | 0.082 | 0.143 |
|  |  |  |  |  |
| Probable HR-HPV | | | | |
| HPV68 | 20 | 0.569 | 0.009 | 0.031* |
|  |  |  |  |  |
| Possible HR-HPV | | | | |
| HPV53 | 17 | 0.691 | 0.002 | 0.017* |
| HPV66 | 15 | 0.460 | 0.084 | 0.143 |
| HPV67 | 10 | 0.601 | 0.066 | 0.140 |
|  |  |  |  |  |
| LR-HPV | | | | |
| HPV6 | 5 | -0.894 | 0.041 | 0.116 |
| HPV11 | 2 | NV | NV | NV |
|  |  |  |  |  |

^a^Classification of low (LR)- and (possible and probable) high-risk (HR-)HPV types included in the Riatol qPCR HPV genotyping assay, according to the HPV classification from the International Agency for Research on Cancer (IARC) 2012 [32]. ^b^Significant relationships between the HPV copies per microliter of DNA extract are indicated by an asterisk when p-values that were adjusted for multiple testing using the false discovery rate (FDR) were smaller than the significance level 0.05. NV: Due to the small amount of samples (n≤2), correlations were not calculated.

**Online Resource 3. Correlation between HPV copies in first-void urine (FVU) as opposed to cervical samples (CS).** The figures show the HPV copies per microliter of DNA extract in FVU (x-axis) and CS (y-axis) on a logarithmic scale for HPV16 (A) and HPV18 (B). Significant relationships between HPV copies are indicated by an asterisk when p-values adjusted for multiple testing using the false discovery rate (FDR) were smaller than the significance level 0.05.

(A) HPV16

(B) HPV18

**Online Resource 4. Disparity in log transformed HPV copies between paired first-void urine and cervical samples.**

| HPV genotype (group)^a^ | N positive samples | |  | Median log HPV copies per  µl of DNA extract (IQR) | | Wilcoxon matched pairs signed rank test (p-value) | FDR-adjusted p-value^b^ | Median log HPV copies per hDNA equivalent (IQR) | | Wilcoxon matched pairs signed rank test (p-value) | FDR-adjusted p-value^b^ |
| --- | --- | --- | --- | --- | --- | --- | --- | --- | --- | --- | --- |
|  | **Cervix** | **First-void urine** |  | **Cervix** | **First-void**  **urine** |  |  | **Cervix** | **First-void urine** |  |  |
| HR-HPV | | | | | | | | | | | |
| HPV16 | 21 | 23 |  | 6.996  (5.847-7.976) | 5.862  (4.138-6.917) | 0.003 | 0.040* | 3.281  (1.988-4.479) | 2.273  (1.476-3.454) | 0.027 | 0.432 |
| HPV18 | 6 | 7 |  | 6.024  (5.436-6.820) | 5.821  (4.952-6.164) | 0.345 | 0.613 | 2.227  (2.161-3.386) | 2.220  (1.887-4.277) | 0.753 | 0.861 |
| HPV31 | 21 | 24 |  | 7.035  (4.455-7.964) | 6.149  (4.862-8.143) | 0.986 | 0.986 | 3.564  (0.699-5.000) | 3.367  (1.740-4.851) | 0.356 | 0.712 |
| HPV33 | 4 | 5 |  | 6.984  (6.663-8.144) | 4.920  (4.443-6.700) | 0.180 | 0.480 | 3.448  (2.629-4.739) | 2.122  (0.778-3.444) | 0.180 | 0.576 |
| HPV35 | 6 | 5 |  | 6.115  (5.471-8.615) | 5.829  (4.010-6.479) | 0.465 | 0.625 | 2.208  (1.789-5.000) | 2.464  (1.003-3.198) | 0.715 | 0.861 |
| HPV39 | 7 | 6 |  | 4.000  (2.631-7.674) | 5.455  (4.702-6.191) | 0.465 | 0.625 | 0.477  (-1.290-4.243) | 2.146  (1.654-3.603) | 0.465 | 0.744 |
| HPV45 | 4 | 8 |  | 7.131  (4.872-8.617) | 5.105  (4.352-5.614) | 0.273 | 0.542 | 3.624  (1.124-4.727) | 1.790  (1.431-2.225) | 0.465 | 0.744 |
| HPV51 | 12 | 18 |  | 6.188  (5.377-7.327) | 5.538  (4.444-6.650) | 0.060 | 0.320 | 2.605  (2.209-3.600) | 2.163  (1.366-3.330) | 0.814 | 0.868 |
| HPV52 | 11 | 7 |  | 6.378  (5.109-8.201) | 5.405  (5.335-6.588) | 0.753 | 0.803 | 2.519  (1.532-4.725) | 2.459  (1.746-3.456) | 0.917 | 0.917 |
| HPV56 | 6 | 6 |  | 6.260  (4.534-7.878) | 5.331  (4.662-6.614) | 0.273 | 0.546 | 2.768  (0.571-4.014) | 2.175  (1.513-3.894) | 0.285 | 0.712 |
| HPV58 | 3 | 5 |  | 8.107  (±0.269)^c^ | 5.622  (5.438-7.249) | 0.109 | 0.349 | 4.302  (±0.429)^c^ | 2.697  (2.415-4.261) | 0.180 | 0.576 |
| HPV59 | 5 | 9 |  | 7.340  (3.805-8.055) | 5.633  (4.143-6.768) | 0.500 | 0.625 | 3.305  (-0.107-4.271) | 2.310  (1.171-3.379) | 0.686 | 0.861 |
|  |  |  |  |  |  |  |  |  |  |  |  |
| Probable HR-HPV | | | | | | | | | | | |
| HPV68 | 10 | 20 |  | 6.366  (5.934-7.314) | 5.349  (4.786-6.034) | 0.005 | 0.040* | 3.069  (2.050-3.807) | 2.238  (1.485-3.034) | 0.059 | 0.472 |
|  |  |  |  |  |  |  |  |  |  |  |  |
| Possible HR-HPV | | | | | | | | | | | |
| HPV53 | 10 | 17 |  | 6.816  (5.955-7.744) | 6.001  (3.092-6.785) | 0.508 | 0.625 | 3.194  (2.530-4.286) | 2.681  (-0.042-4.289) | 0.314 | 0.712 |
| HPV66 | 10 | 13 |  | 7.395  (6.077-8.619) | 7.141  (5.617-7.715) | 0.575 | 0.657 | 3.715  (2.477-4.923) | 4.311  (2.270-4.641) | 0.612 | 0.861 |
| HPV67 | 4 | 9 |  | 7.070  (3.495-8.117) | 3.573  (3.083-6.436) | 0.109 | 0.349 | 3.375  (-0.405-4.637) | 0.749  (-0.094-3.083) | 0.109 | 0.576 |
|  |  |  |  |  |  |  |  |  |  |  |  |
| LR-HPV | | | | | | | | | | | |
| HPV6 | 4 | 2 |  | NV  (6.106-8.447)^c^ | NV  (5.599-7.890)^c^ | NV | NV | NV  (2.583-4.572)^c^ | NV  (2.838-4.650)^c^ | NV | NV |
| HPV11 | 2 | 2 |  | NV  (4.425-6.677)^c^ | NV  (4.104-5.066)^c^ | NV | NV | NV  (0.477-3.154)^c^ | NV  (1.646-2.305)^c^ | NV | NV |
|  |  |  |  |  |  |  |  |  |  |  |  |

^a^Classification of low (LR-) and (possible and probable) high-risk (HR-) HPV types included in the Riatol qPCR HPV genotyping assay, according to the HPV classification from the International Agency for Research on Cancer (IARC) 2012 [32]. ^b^Significantly different median log HPV copies per µl DNA extract or per hDNA equivalent between paired samples are indicated by an asterisk (Wilcoxon matched pairs signed rank test), when p-values adjusted for multiple testing using the false discovery rate (FDR) are smaller than the significance level 0.05). ^c^Mean (± SE: standard error) for n=3. NV: when n≤2 the range (min-max) was reported^c^ and statistical analyses were not performed.

**Online Resource 5. Descriptive analysis of the acceptability of first-void urine sampling using a first-void urine collection device in a cervical cancer referral population.**

| **Question asked with corresponding answer** | **Number of responses/number of women questioned (%)** | **Number of correctly filled in responses (valid %)** | **Number of responses (valid %)** | | | |
| --- | --- | --- | --- | --- | --- | --- |
|  |  |  | **Fully**  **agree** | **Rather**  **agree** | **Rather disagree** | **Fully**  **disagree** |
| **Do you think that this method of urine collection with the Colli-Pee^®^ device, is a good method to perform at-home?** | | | | | | |
| Number of responses | 122/124 (98.39) | 122/122 (100.00) | 100/122 (81.97) | 15/122 (12.30) | 3/122 (2.46) | 4/122 (3.28) |
| No answer was given | 2/124 (1.61) | - | - | - | - | - |
|  |  |  |  |  |  |  |
| **Do you think that this method of urine collection with the Colli-Pee^®^ device, is a good method to perform during a consultation at the general practitioner/in the restrooms of the clinic?** | | | | | | |
| Number of responses | 120/124 (96.77) | 120/120 (100.00) | 62/120 (51.67) | 19/120 (15.83) | 27/120 (22.50) | 12/120 (10.00) |
| No (ambiguous) answer was given^a^ | 4/124 (3.23) | - | - | - | - | - |
|  |  |  |  |  |  |  |
| **Which method do you prefer?** |  |  |  |  |  |  |
| Urine collection using the Colli-Pee^®^ device^b^ | 83/124 (66.94) | 83/113 (73.45) | - | - | - | - |
| Smear collected by a doctor/gynaecologist | 24/124 (19.35) | 24/113 (21.24) | - | - | - | - |
| No preference^c^ | 6/124 (4.84) | 6/113 (5.31) | - | - | - | - |
| No answer was given | 11/124 (8.87) | - | - | - | - | - |
|  |  |  |  |  |  |  |

^a^Three women did not provide an answer to the question, while one woman had no preference. ^b^Five out of 83 women preferred the Colli-Pee^®^ method if it were as reliable as a smear collected by a doctor/gynaecologist. One woman only preferred this method if it could be performed at-home. Another woman brought up the issue that no questions could be asked to the doctor/gynaecologist. ^c^Although ‘no preference’ was not an option in the questionnaire, five women wrote down that they did not have a preference between the two methods or checked both boxes. One woman preferred yearly first-void urine collection with the Colli-Pee^®^ and biyearly smear collection by the doctor/gynaecologist.
